# Seroepidemiology of Hepatitis B and C Viruses in the General Population of Burkina Faso

**DOI:** 10.1155/2014/781843

**Published:** 2014-08-05

**Authors:** Issoufou Tao, Tegwindé R. Compaoré, Birama Diarra, Florencia Djigma, Theodora M. Zohoncon, Maléki Assih, Djeneba Ouermi, Virginio Pietra, Simplice D. Karou, Jacques Simpore

**Affiliations:** ^1^Centre de Recherche Biomoléculaire Pietro Annigoni (CERBA), BP 364, Ouagadougou 01, Burkina Faso; ^2^Laboratoire de Biologie Moléculaire et de Génétique (LABIOGENE), Université de Ouagadougou, BP 7021, Burkina Faso; ^3^Centre Médical Saint Camille (SCMC), BP 364, Ouagadougou 01, Burkina Faso; ^4^École Supérieure des Techniques Biologiques et Alimentaires (ESTBA-UL), Université de Lomé, BP 1515, Togo

## Abstract

*Objectives.* In Burkina Faso, few studies reported the prevalence of HBV and HCV in the general population. This study aimed to evaluate the prevalence of hepatitis B and C viruses in the general population and to determine the most affected groups in relation to the risk factors associated with the infection. *Method.* A voluntary testing opened to anyone interested was held at Saint Camille Medical Centre in Ouagadougou. Rapid tests were carried out on 995 persons who voluntarily answered a range of questions before the venous blood sampling. *Results.* The results revealed that the antigen HBs carriers in the general population represented 14.47% (144/995) and the prevalence of HCV was 1.00% (10/995). The difference between HBV's prevalence in men (18.58%) and that in women (11.60%) was statistically significant (*P* = 0.002). The most affected groups were undergraduated students (19.57%) and persons working in the informal sector (15.98%). The least affected group was high level students (8.82%). *Conclusion.* Burkina Faso is a country with a high prevalence of HBV, while the incidence of HCV is still low in the general population. Therefore, more campaigns on the transmission routes of HBV and HCV are needed to reduce the spread of these viruses in sub-Saharan Africa.

## 1. Introduction

According to the World Health Organization, more than 240 million people are infected with the hepatitis B virus (HBV) worldwide, and the majority is living in the developing countries [1]. Yearly, there are more than 600000 deaths due to the complications related to the infection. HBV's association with liver diseases, such as the primary liver carcinoma and cirrhosis, is clearly established [2, 3]. The HBV prevalence is around 15% in Southeast Asia [4]. In Africa, the virus is highly endemic [5]. Because of its high HBV prevalence, Burkina Faso has been classified by WHO in 2002 as an area of high endemicity [6].

Hepatitis C virus (HCV) in Burkina Faso causes about 900 deaths per year. This virus is also a major risk factor for the liver cancer [7]. HBV and HCV are easily transmissible through sexual, parenteral, and vertical routes [8]. Several behavioral, environmental, and cultural factors may also be responsible for their infections [9]. In Africa, after the vertical and the sexual transmissions, HBV and HCV infections are due to cultural practices (levirate, sorority, sexual rituals, scarification, piercing, and tattoos) or medical surgeries [10, 11]. HBV and HCV are easily transmitted than the Human herpes virus 8 (HHV-8) [12]. They are even cited as risk factors associated with the infection by HHV-8 and HIV [13, 14].

In Burkina Faso, many studies have reported different prevalence for HBV and HCV among target groups. In fact, authors reported that 12.1% patients in the health district of Nanoro [15], 18% among blood donors of Nouna, 11% among blood donors of Ouagadougou [16], and 9.3% among pregnant women of Ouagadougou [14] were infected by HBV. For the HCV infection, studies report 4.4% among blood donors of Regional Center of Blood Transfusion of Ouagadougou [17] and 0.6% among health professionals in the district of Nanoro [15]. However, there are very few studies on the prevalence of HBV and HCV across the general population of Burkina Faso. HBV constitutes a public health problem; Burkina Faso's Ministry of Health adopted strategies such as strengthening the prevention of infections in the health care facilities and blood safety measures, as well as the integration of HBV vaccine through the expanded program on immunization (EPI) [15]. This study aimed to (1) evaluate the seroprevalence of HBV and HCV in the general population of Burkina Faso, (2) determine the most affected groups by the infections with hepatitis B and hepatitis C, and (3) study the risk factors associated with the HBV and HCV infection.

## 2. Methodology

The study was conducted during an awareness campaign against hepatitis organized by “SOS Hépatites Burkina.” “SOS Hépatites Burkina” is an association of professionals that educates people about hepatitis. The campaign and the study took place in the Saint Camille Medical Centre in Ouagadougou. It involved 995 people composed of 586 women and 409 men. Testing was free and voluntary, and sampling was preceded by individual counseling. The subjects responded to a range of questions concerning their age, marital status, criminal record, profession, serostatus for HIV, intravenous drugs use, and health history. Biological parents or relatives have given their consent for infants. The results were made available during single post test counseling. The presence of the HBs antigen and anti-VHC antibodies were both determined by the rapid tests ABON.

## 3. Ethical Issues

The study was approved by the institutional ethics committee of the Centre for Biomolecular Research Pietro Annigoni and that of Saint Camille Medical Centre. All the persons who participated in this study gave their informed consent.

## 4. Data Analysis

Statistical analysis was performed with Epi Info version 6 and SPSS version 20 software. *P* values ≤ 0.05 were considered significant.

## 5. Results

The surface antigen HBs and the anti-HCV antibodies screening concerned 995 individuals of which 586 (58.89%) were women and 409 (41.10%) were male. The ages ranged from 8 to 75 years (with a mean of 41.5 ± 12.6 years). According to the marital status, 53.80% (535/995) were single, 439 (44.10%) were married, and the rest were divorced or widowed. For the professional status, the majority was civil servants, followed by the informal sector workers, the undergraduate students, and the high level students (Table 1). With regard to the professional status, the most affected groups by HBV infection were undergraduate students and individuals working in the informal sector.  Table 2 displays the sociocultural practices in relation to HBV infection. According to the table, 68.73% of the population was circumcised, or doing piercing, tattooing, or scarification, or had a genital mutilation; 35.60% had unprotected sex. In relation to the clinical background, 14.40% of the studied population had undergone medical surgery and 13.60% had at least once been hospitalized (Table 3). The present results also showed that the mature age, marital status, gender, and job insecurity (informal sector) are some risk factors for HBV infection. Indeed, 15.52% of married people were HBV carriers against 14.95% for singles. Analysis done on gender showed a significant difference (*P* = 0.002) between the rates of infection among men (28.58%) and women (11.60%). The most affected age class was 31–40 years with an infection rate of 16.33%. The least affected age group was 41−50 years with an infection rate of 11.27% (Table 4). There were no significant differences between HBV prevalence in people who benefited from a blood transfusion and those who have not (*P* = 0.81) nor between those having undergone surgery and those who have not (*P* = 0.25). However, there was a significant difference between the prevalence of HBV among genital mutilated or circumcised people (16.18%) and uncircumcised or genital nonmutilated individuals (11.22%): *P* = 0.04. In general, cultural practices appeared to be risk factors associated with HBV infection. Only 1.00% of the population was concerned with the infection of HCV. Civil servants were the most affected (2.03%), while individuals from the informal sector represented the least affected group (0, 49%).

## 6. Discussion

This study aimed to assess the prevalence of HBV in the general population of Ouagadougou. Data analysis confirmed the high prevalence (14.5%) of hepatitis B and the low one of hepatitis C (1.00%) in the general population of Burkina Faso. The very low prevalence of HCV did not allow us to draw correlations and discuss it. HBV prevalence is in the range of 10–17% reported in adults in Nigeria [18, 19]. These results also show a higher HBV prevalence than in the target groups as reported by other studies. Indeed, Pietra et al. [15] reported a prevalence of HbsAg of 12.1% in the health professionals of Nanoro district; Collenberg et al. in 2006 [16] reported an HBV's prevalence of 14.3% (Nouna) and 17.3% (Ouagadougou) in blood donors and pregnant women. However, a prevalence of 12.9% was recorded in 2013 in blood donors of the National Blood Transfusion Center of Burkina Faso [20]. Several studies agreed that HBV prevalence is lower in rural than in urban areas [2, 16, 21]. The significant difference (*P* = 0.002) between HBV infections in men and women reported in this study is consistent with the results obtained by Deng et al. [22] in 2013 in China (6.54% versus 3.87%) and Makuwa et al. [21] in Gabon in 2009 (16.2% versus 9.9%). This study reports an HBV/AIDS coinfection of 22.22%. This is a common coinfection, given the fact that the two viruses share the same transmission routes [11, 23, 24]. This study also reports an HBV infection in 21.42% of children under 12 years of age who are born from HIV and HBV positive mothers. At this stage, the study cannot demonstrate the evidence of a vertical transmission or a horizontal infection. In fact, some traditional practices could explain the high prevalence of HBV in children, particularly the mothers using saliva to heal baby wound. It is in this sense that Kiire confirmed that horizontal transmission is the main route of transmission of HBV in babies [25]. However, vertical transmission probably plays an important role, as in Burkina no action (HBV screening during pregnancy, vaccination at birth) is taken to fight against it. The lack of significant difference in the prevalence of HBV among people when taking into account their health background (blood transfusion, surgery, and hospitalization) can be explained by the improvement of blood safety and the health management system in Burkina Faso. In fact, HIV, hepatitis B and hepatitis C, and the bacterium* Treponema pallidum subspecies pallidum* are routinely detected in blood donations [20]. However, the prevalence of 16.21% of HBV among transfused persons against 14.40% in nontransfused shows that the contamination by residual risk of blood transfusion remains. The age group of 30–40 years of age is the most affected (16.33%), followed by 20 to 30 years (15.9%). These results show that young people are most affected by HBV infection. These results are similar to those of Makuwa et al., [21] who reported a prevalence of 22.22% among young men in the same age group in urban areas of Gabon. The low prevalence of individuals in the age group above 50 years of age could indicate that several people in this group might have died from cirrhosis or liver cancer due to lack of medical care. We note a higher prevalence of HBV (15.52%) among married individuals compared to single individuals (14.95%). This study also reports a high prevalence of HBV among widowed (28.57%). The lowest prevalence occurs among the high school student's group (8.82%). Undergraduate students are the most affected group with a prevalence of 19.57%. This could be explained by the fact that they are at the prime of life and are likely to have risky sexual behaviors. Finally, this study reported a high prevalence (16.00%) of HBV in persons who were circumcised, or had a genital mutilation, or had a piercing, a tattoo, or a scarification. This confirms that these cultural practices are risk factors associated with HBV infection [26].

## 7. Conclusion

This study reports a high prevalence of HBV infection in Burkina Faso. Many people do not have information on the importance of vaccination against HBV as primary prevention. They also ignore the support possibilities of medical chronic hepatitis. Better organization and increased awareness campaigns on HBV and HCV will reduce their prevalence. Moreover, the reduction of the HBV vaccine cost will lower the spread of the hepatitis B virus. In the short term, we suggest working with control structures against HIV to organize forums to raise awareness on HIV, HBV, and HCV.

## Figures and Tables

**Table 1:** Sociodemographic data in relation to the HBV infection throughout the population.

Characteristics	Profession						Marital status			
	Civil servant	Informal sector	High school students	Undergraduate students	Housewives	Trader	Single	Married	Widow	Divorced
Total	211	169	155	152	112	52	535	439	14	7
HBV (+) number %	35 14.22	32 15.98	15 8.82	37 19.57	17 13.17	8 13.33	80 14.95	59 15.52	4 28.57	1 14.28
OR (95% IC)	—	—	—	—	—	—	—	—	—	—
*P* values				0.10			0.43			

**Table 2:** The sociocultural practices in relation to the HBV infection in the population.

	Cultural practices						Prison	
	Transcutaneous examination or acupuncture		Excision, circumcision, and tattooing		Unprotected sex			
	+	−	Circumcised and female mutilated genital	Piercing, scarification, and tattooing	+	−	+	−
Total	37	958	649	34	354	641	04	991
HBV (+) *N* (%)	03 8.10	141 14.71	105 32.28	04 11.76	54 15.25	90 14.04	0.00	144 14.53
OR (95% IC)	1	1.5 0.1–1.6	—		1.1 0.7–1.5	1	—	
*P* values	0.34		—		0.63		—	

**Table 3:** Clinical background in relation to the HBV infection in the population.

	Blood transfusion		Surgery		Hospitalized		HBV mothers		HIV		HCV	
	+	−	+	−	+	−	+	−	+	−	+	−
Total	37	958	143	852	135	860	28	967	9	986	10	985
HBV (+) *N* (%)	6 16.21	138 14.40	16 11.18	128 15.02	21 15.55	123 14.30	6 21.42	138 14.27	2 22.22	142 14.4	0 0.00	144 14.21
OR (95% IC)	1.15 0.4–2.8	1	1	0.7 0.4–1.2	1.1 0.6–1.8	1	1.6 0.1–1.6	1	1.6 0.3–8.2	1	—	
*P* values	0.81		0.25		0.69		0.27		0.62		—	

**Table 4:** HBV infection by gender and age classes.

	Gender		Age classes				
	Male	Female	<20	21–30	31–40	41–50	≥50
Total	409	586	155	358	251	133	98
HBV (+) (%)	76 18.58	68 11.60	19 12.25	57 15.92	41 16.33	15 11.27	12 12.24
OR (95% IC)	1.7 (1.2–2.4)	1	—	—	—	—	—
*P* values	0.002		—				
